# Expanding the Horizons of Pre-Transplant Renal Vascular Assessment Using Ex Vivo Perfusion

**DOI:** 10.3390/cimb45070345

**Published:** 2023-06-29

**Authors:** Carolina Campos Pamplona, Cyril Moers, Henri G. D. Leuvenink, L. Leonie van Leeuwen

**Affiliations:** Department of Surgery—Organ Donation and Transplantation, University Medical Center Groningen, University of Groningen, Hanzeplein 1, 9713 GZ Groningen, The Netherlands; c.campos.pamplona@umcg.nl (C.C.P.); c.moers@umcg.nl (C.M.); h.g.d.leuvenink@umcg.nl (H.G.D.L.)

**Keywords:** kidney, transplantation, ex vivo perfusion, vasculature, endothelial cells, homeostasis, viability assessment

## Abstract

Recently, immense efforts have focused on improving the preservation of (sub)optimal donor organs by means of ex vivo perfusion, which enables the opportunity for organ reconditioning and viability assessment. However, there is still no biomarker that correlates with renal viability. Therefore, it is essential to explore new techniques for pre-transplant assessment of organ quality to guarantee successful long-term transplantation outcomes. The renal vascular compartment has received little attention in machine perfusion studies. In vivo, proper renal vascular and endothelial function is essential for maintaining homeostasis and long-term graft survival. In an ex vivo setting, little is known about vascular viability and its implications for an organ’s suitability for transplant. Seeing that endothelial damage is the first step in a cascade of disruptions and maintaining homeostasis is crucial for positive post-transplant outcomes, further research is key to clarifying the (patho)physiology of the renal vasculature during machine perfusion. In this review, we aim to summarize key aspects of renal vascular physiology, describe the role of the renal vasculature in pathophysiological settings, and explain how ex vivo perfusion plays a role in either unveiling or targeting such processes. Additionally, we discuss potentially new vascular assessment tools during ex vivo renal perfusion.

## 1. Introduction

At present, kidney transplantation is the best treatment for end-stage kidney disease [1]. However, the supply of donor kidneys for transplantation does not match the demand of patients waiting for a new organ. In 2020, worldwide data showed that every year, about 15% of patients die on the waiting list for a kidney transplant, 15–20% of deceased donor kidneys are declined for transplantation, and around 30% of transplanted kidneys do not show acceptable outcomes [2,3]. In an attempt to sustain the need for donor grafts, the acceptance of suboptimal kidneys, such as those recovered from extended criteria donors (ECD) and circulatory death donors (DCD), is becoming more common. ECD donors are donors over 60 years of age or over 50 with vascular comorbidities, as donor age is reported to be the single independent risk factor for graft survival [4]. DCD donors are one of the two types of post-mortem donors who have sustained irreversible brain injury but do not meet formal brain death criteria. These grafts suffer a higher amount of injury when compared with brain-dead donor grafts due to the infliction of a higher warm ischemic injury during procurement [5]. Unfortunately, the lower quality of these organs introduces higher risks of delayed graft function (DGF) and primary non-function (PNF) compared to grafts from standard criteria donors and living donors [6,7,8].

Eurotransplant statistics from 2021 show that, out of 4027 kidney transplantations in the Eurotansplant area, around 73.4% of the organs were obtained from deceased donors, and out of all the kidneys offered, only around 88.4% were transplanted. These data suggest that a substantial number of kidneys could potentially be accepted for transplantation. To safely accept a higher number of lower-quality organs, clinicians and surgeons would benefit from reliable and objective pre-transplant organ viability assessments that ensure positive post-transplant outcomes. However, none of the current tools can successfully and independently predict post-transplant outcomes. This means there is still no biomarker that correlates with renal viability, making the prediction of transplant outcomes, such as immediate function, DGF, and PNF, very difficult [9]. Therefore, it is essential to explore new tools and techniques for pre-transplant assessment of organ quality to guarantee successful transplantations and positive long-term outcomes.

In recent years, immense efforts have focused on improving the preservation of (sub)optimal donor organs by means of ex vivo perfusion, which entails a controlled flow of perfusion solution through isolated kidney grafts, enabling an extended preservation time and the opportunity for organ reconditioning and viability assessment prior to transplantation [10,11,12]. Hypothermic (1–10 °C) machine perfusion (HMP) is increasingly used as a clinical preservation method for deceased donor kidneys. It is preferred over static cold storage (SCS), as it keeps the organ homogeneously cooled, constantly recirculates a protective preservation solution throughout the kidney, and decreases vascular resistance during preservation and at reperfusion. Ultimately, this technique has been shown to improve transplant outcomes and reduce the incidence of DGF [13,14,15]. However, during HMP, it is not possible to assess renal functionality due to its hypometabolic state [16]. Normothermic machine perfusion (NMP) is a relatively new preservation technique in which the kidney is warmed back to normothermic temperatures (35–37 °C), restoring metabolism and thus creating a pre-transplant window to monitor kidney function and viability in a close to physiological setting [3,9,10,17]. This platform also enables the administration of drugs and other treatments [17,18,19,20]. Hence, NMP opens doors to extending organ preservation time, real-time evaluation of kidney function, and active organ reconditioning [18,21,22].

Different from other organs, a reliable and independent pre-transplant evaluation biomarker for renal viability ex vivo has not yet been identified, although it is widely studied [9]. For example, multiple studies have investigated nephron function and injury, perfusate composition, and immune response [9,17,23,24,25,26,27,28,29,30,31,32]. In contrast, the renal vascular compartment has hardly been studied in a machine perfusion setting. We know that, in vivo, proper renal vascular and endothelial function is essential for maintaining homeostasis and—in a transplantation environment—long-term graft survival [33]. Injury to the endothelial monolayer is the first step in a cascade of disruptions, such as increased vascular permeability, impaired vasoactive capacity, leukocyte adhesion, inflammation, edema, thrombosis, and turbulent flow. It has been associated with multiple disorders such as systemic sclerosis, sepsis, diabetes mellitus, cardiovascular disease, pulmonary arterial hypertension, ischemia-reperfusion injury (IRI), and cancer [34,35,36].

However, in an ex vivo setting, little is known about vascular viability, its implications on organ suitability for transplant, and its related complications. During perfusion, flow and vascular resistance are the most common parameters described to report vascular function, but their real diagnostic value has not yet been established [9]. Seeing that the vascular compartment is of high importance to homeostasis and good post-transplant outcomes, taking a step back and looking into basic physiology and conducting further research is key to clarifying the (patho)physiology of the renal vasculature during machine perfusion. To the authors’ knowledge, no one has yet reviewed the importance of viability assessment of the renal vascular compartment in an ex vivo setting. Therefore, we decided to investigate its potential merits. In this review, we first summarize key aspects of renal vascular physiology. We then describe the role of the renal vasculature in transplantation-related pathophysiological settings and how ex vivo perfusion plays a role in either unveiling or targeting such processes. Lastly, we discuss possible applications of vascular assessment tools during ex vivo renal perfusion.

## 2. Renal Structure and Homeostasis

### 2.1. Vascular Structure and Function of the Kidney

**Figure 1:**
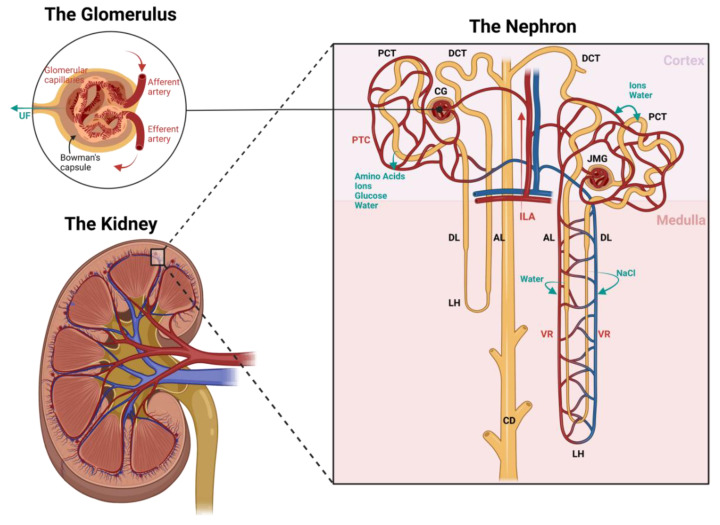
Schematic image of the renal (vascular) structure. Black labels indicate the nephron structures, red labels indicate the vascular structures, and blue labels indicate the filtration processes. Blood flow is directed to an area of glomeruli via interlobular arteries (ILA). Glomerular capillary flow—which is surrounded by Bowman’s capsule—is flanked by two resistance vessels: inflow via afferent arteries and outflow via efferent arteries. In this structure, the ultrafiltrate (UF) is formed. The distal end of the efferent artery can follow one of two paths depending on the location of the glomerulus: if the glomerulus resides in the outer cortex (CG), the effluent blood will run alongside the proximal (PCT) and distal convoluted tubules (DCT) into the peritubular capillaries (PTC), and if the glomerulus resides in the juxtamedullary region (JMG), the effluent blood will run parallel to the loops of Henle (LH) into the vasa recta (VR). The ultrafiltrate formed in the glomeruli flows past the PCT to re-absorb amino acids, ions, glucose, and water, and as it runs through the descending (DL) and ascending limb (AL) of the Loop of Henle, it continues to re-absorb water and sodium chloride into the circulation to control the urine osmolarity. Finally, the urine is fine-tuned in the DCT by exchanging water and ions, and flows into the collecting duct (CD), then to the renal pelvis, and down to the bladder via the ureter, where it is stored until urination. Image created with BioRender.

To understand the structure and physiology of the renal vasculature, it is important to acknowledge the main function of the kidneys—filtration. The kidneys receive about 20–25% of the cardiac output, although they only constitute 0.4% of total body weight [37]. This high rate of blood flow is critical for the homeostatic regulation of water and ion content in the blood. Therefore, well-functioning renal circulation is of utmost importance. The kidney is one of the organs with the most unique vascular structures and diversified cell types, including different endothelial cell (EC) populations. This is due to the necessity of selective permeability for the transportation of different compounds, but also to enable survival in environments with different oxygen contents and osmolarities [38,39]. In the following topics, we will describe the general (vascular) structure of the kidney, as shown in Figure 1.

#### 2.1.1. The Nephron

The kidney consists of about one million microscopic structural and functional units called nephrons. As seen in Figure 1, the nephron is composed of the glomerulus, proximal convoluted tubule, loop of Henle, distal convoluted tubule, and collecting duct [38,40]. A vast network of glomeruli resides in the cortex, the most peripheral and oxygen-rich part of the kidney. Some in the outer cortex and some in the juxtamedullary region. Oxygenated blood passes through the glomeruli via a glomerular capillary network and is roughly filtered from electrolytes and water. In Bowman’s capsule, this ultrafiltrate, which will eventually become urine, flows into the proximal convoluted tubule (PCT), which functions to re-absorb and transport water and electrolytes back into circulation [38,39]. The PCT penetrates the outer and inner medullary zone in a loop structure referred to as the loop of Henle [38].

The medullary region of the kidney receives only around 10% of the kidney’s total blood flow, resulting in a lower oxygen content in the tissue. The resulting oxygen gradient between the cortex and the medulla is essential to maintaining an osmotic gradient that maximizes filtration capacity [40]. In this zone, the loop of Henle is responsible for re-absorbing solutes, especially sodium, therefore regulating essential changes in urine osmolarity. Near the end of the nephron and back in the cortex, the distal convoluted tubule (DCT) is responsible for further optimizing urine composition, and a cluster of transporters aids in solute re-absorption. The final part of each nephron is composed of the collecting duct, where the last fine-tuning of the urine takes place by regulating hydrogen and bicarbonate secretion. The final urine product is then transported to the pelvis of the kidney, which directs it to the bladder via the ureter [38,39].

#### 2.1.2. The Vasculature

As filtration, re-absorption, and secretion occur throughout the structures of the kidney, an organized network of blood vessels is key for optimal functioning. Following Figure 1, blood enters the kidney via a series of branching arteries originating from the main renal artery. These branches are directed to the cortex via interlobular arteries and into the glomeruli network. Glomerular capillary flow is flanked by two resistance vessels: inflow via afferent arteries and outflow via efferent arteries. Each of these capillaries consists of a different vascular composition to ensure optimal filtration. Afferent arterioles consist of a continuous smooth muscle cell (SMC) layer and are responsible for driving the blood flow rate. Glomerular capillaries are built with continuous, fenestrated endothelial cells capable of selectively filtrating macromolecules based on size and charge. These ECs are well known to synthesize nitric oxide (NO) and endothelin-1 (ET-1) and express vascular endothelial growth factor (VEGF) [40]. Efferent arterioles, in contrast, possess a limited discontinuous layer of SMCs to regulate the filtration fraction, and their distal end can follow one of two paths depending on the location of the glomerulus: if the glomerulus resides in the outer cortex, the effluent blood will run alongside the proximal and distal convoluted tubules (peritubular capillaries), and if the glomerulus resides in the juxtamedullary region, the effluent blood will run parallel to the loops of Henle (vasa recta) [40]. The peritubular capillaries (PTCs), located close to both convoluted tubules, are responsible for delivering blood to the tubules and acting as a transporter of the re-absorbed ions, amino acids, glucose, and water [38,39,40,41]. The vasa recta, running parallel to the loops of Henle and collecting tubules, are the sole blood supply of the medullary region. Their function is to control blood flow through the medulla by detecting vasoactive signals from pericytes [40].

Pericytes are a specific type of perivascular mural smooth muscle cell that encircles ECs, and their role is to maintain vessel stabilization and regulate fibrosis, EC response, and angiogenesis [40,42]. These cells are responsible for the regulation of peritubular capillaries and EC stability [42,43,44,45]. Different parts of cortical and medullary vessels present variations in pericyte and EC composition, enabling regulation of vessel diameter and, therefore, blood flow [40]. Studies have suggested the presence of vasoactive feedback loop stimuli in juxtamedullary resistance vessels that allow the renal medulla to regulate its own perfusion rate, with the intention of directing the majority of the blood flow to the cortex [40,43,44,45].

Considering the main function of the kidneys is to act as a blood filter to maintain osmotic balance and to excrete waste products via the urine, it is of great importance that the vasculature and, therefore, endothelial cells are viable and well-functioning.

### 2.2. The Importance of Renal Circulation in Homeostasis

The renal vessels play a key role in kidney function, as it is responsible for maintaining homeostasis. Homeostasis is described as the maintenance of vascular function over time, including the adaptation to environmental changes [46]. Healthy endothelial cells are responsible for regulating antioxidant, vasoactive, coagulatory, and inflammatory responses. Irreparable damage to this monolayer of cells is directly correlated with multiple pathologies, such as peripheral arterial disease, cardiovascular disease, chronic kidney disease, and renal fibrosis [35].

The integrity of the inter-endothelial barrier and its cell-to-cell communication is primarily composed of tight, gaps, and adherent junctions. These transmembrane proteins are responsible for assuring controlled permeability of plasma, proteins, and cells. When there is endothelial dysfunction, a vicious circle of injury and inflammation takes place: increased permeability allows hemoconcentration, which, in turn, increases endothelial-leukocyte interactions, sheer stress, and coagulation and, therefore, promotes injury [33,35,36,40,41,47,48].

In addition to cell-to-cell integrity, all endothelial cells possess a delicate luminal membrane that adds a layer of protection and homeostasis control, also known as the glycocalyx. This structure has been a focus of research in recent years as it is of great importance for vascular function. Some of its functions involve sheer stress-dependent production of nitric oxide, maintaining anticoagulation properties, modulating inflammatory cell adhesion, and playing a role in balancing fluid exchange [49,50]. When renal injury occurs, the glycocalyx is the first to be affected, and its components, such as syndecan-1, heparan sulfate, and hyaluronic acid, are shed into the bloodstream. This effect has been identified in response to multiple disorders in rodent, porcine, and human models, and in most cases, the circulating levels of syndecan-1 and heparan sulfate are proportionally related to the amount of injury caused to the glycocalyx [50].

## 3. Vasoactivity in a Nutshell

Endothelial cells are of great importance to renal blood flow regulation in response to sympathetic, hypoxia, and sheer stress signaling [48]. Vasoactivity controls the blood flow rate for higher or lower urine concentration and drives oxygen delivery between the cortex and the medulla. The slightest dysfunction in this balance can inflict hypoxia in the medullary region. Therefore, in communication with SMCs and pericytes, healthy ECs selectively stimulate vasoactivity to control blood flow and inhibit circulating cell adhesion and platelet aggregation. Injured ECs can, as a result, induce a vasoconstrictive, pro-inflammatory, and procoagulant phenotype in the kidney [33,36,40].

**Figure 2:**
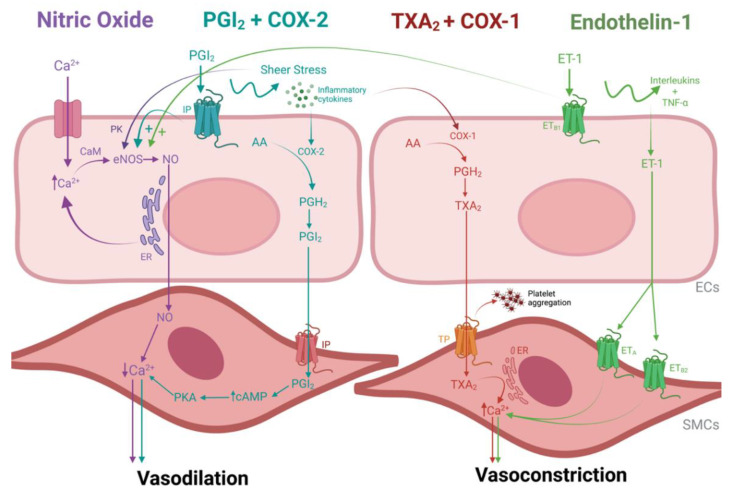
Schematic image of vascular contractility pathways in the endothelial cells (ECs). Purple labels indicate the nitric oxide (NO) signaling cascade, blue labels indicate the prostacyclin (PGI_2_) cascade, red labels indicate the thromboxane (TXA_2_) cascade and green labels indicate the endothelin-1 (ET-1) cascade. The main pathway of NO production starts with the activation of the endothelial enzyme nitric oxide synthase (eNOS) by an intracellular influx of calcium (Ca^+^) and/or an agonist signal to release Ca^2+^ from the endoplasmic reticulum (ER). Ca^2+^ is then modified by calmodulin (CaM) so that it can bind to eNOS, which then results in the production of NO. Once Ca^2+^ levels are generally depleted and more long-term vasodilation is needed, an alternative pathway is activated to stimulate NO production directly. Namely, the phosphorylation of eNOS via protein kinases (PK) in response to sheer stress on the cell surface. Both pathways culminate in sending NO to the smooth muscle cells (SMCs), which stimulates a decrease in Ca^2+^ levels and promotes relaxation; The PGI_2_ pathway, a vasodilatory pathway that compensates for low NO levels, is mediated and catalyzed by cyclooxygenase enzyme 2 (COX-2), which is activated by sheer stress and ECs are damaged and exposed to inflammatory cytokines. The conversion of arachidonic acid (AA) into prostaglandin H_2_ (PGH_2_) by COX-2 is the initial step to synthesize PGI_2_. PGI_2_ then activates platelets and SMCs by binding to a prostacyclin receptor (IP). In response, SMCs produce adenosine monophosphate (cAMP) and activate protein kinase (PKA), which, in turn, relaxes the SMCs in the same way as NO. The binding of circulating PGI_2_ to their EC IP receptor can also promote vasodilation by stimulating eNOS; In contrast, the production of TXA_2_ induces vasoconstriction and platelet aggregation. Cyclooxygenase enzyme 1 (COX-1) converts AA into PGH_2_, which stimulates the isomerization of TXA_2_. In sequence, TXA_2_ binds to thromboxane-prostanoid receptors (TP) located in SMCs and platelets, therefore inducing platelet aggregation. Vasoconstriction by TXA_2_ can also occur by stimulating CA^2+^ release by the endoplasmic reticulum in SMCs. Lastly, the ET-1 pathway is regulated by inflammatory cells (interleukins and TNF-α) stimulation and reduction in NO and PGI_2_ levels. ET-1 receptors are present both in ECs (ET_B1_) and SMCs (ET_A_ and ET_B2_). When SMC ET-1 receptors are activated, there is an influx of calcium due to the opening of the Ca^2+^ channels, causing vasoconstriction in a similar way as TXA_2_. In contrast, activation of ET_B1_ receptors in ECs causes vasodilation by stimulating the release of PGI_2_ and NO. When endothelial dysfunction occurs, EC ET-1 receptors are downregulated, and SMC ET-1 receptors are upregulated, therefore enhancing a vasoconstrictive environment. Image created with BioRender.

To add even more complexity, the kidney is an organ with one of the most diversified EC populations. Different parts of the renal vasculature have different cellular arrangements and present production or sensitivity to different vasoactive and regulatory signaling, making vasoactive control more complex. Pericytes respond to vasoactive signals from ECs and control blood flow to the medulla by regulating the dilation/contraction of the vasa recta. Each endogenous stimuli has its own activation/inhibition pathways, as illustrated in Figure 2, and these need to be tightly regulated to maintain homeostasis and functional renal flow for oxygenation and filtration purposes [40].

### 3.1. Vasodilation

#### 3.1.1. Nitric Oxide

Nitric oxide (NO) is an endothelial-dependent vasodilator produced by endothelial cells, which also has protective effects towards leukocyte activation and adhesion. NO production derives from endothelial enzyme nitric oxide synthase (eNOS) activation, either from an increase in intracellular levels of Ca^2+^ or as a response to sheer stress on the cell surface. Either way, the produced NO is diffused to the SMC/pericytes to stimulate a decrease in tension, thus inducing vasodilation [48,51,52].

In the presence of endothelial dysfunction, the basal dilatory tone is compromised, sheer stress on the cell surface is increased, and further damage occurs. An early rabbit study by Rees et al. [53] reported that the in vivo systemic blood pressure increases due to vasoconstriction when blocking NO production by administration of N^G^ monomethyl-L-arginine (L-NMMA)—a physiological precursor of NO. In vivo administration of L-NMMA followed by ex vivo perfusion of the thoracic artery successfully reversed the compound’s inhibition after infusion of L-arginine, therefore highlighting that NO is crucial to maintaining basal vasodilation.

#### 3.1.2. Prostacyclin and Cyclooxygenase Enzyme 2

Prostacyclin-mediated vasodilation is a separate pathway that can help compensate for low NO levels but also has its own protective effects regarding leukocyte adhesion. This prostanoid pathway is mediated and catalyzed by cyclooxygenase enzyme (COX)-2, which becomes activated when there is sheer stress and the ECs are damaged and exposed to inflammatory cytokines. COX-2 drives the initial step to synthesize prostacyclin (PGI_2_). PGI_2_ then activates platelets and induces relaxation of SMCs in the same way as NO. However, it is important to note that the NO and PGI_2_ pathways are not dependent on each other, meaning that if one is blocked, there might still be residual compensatory vasodilation from the other [48,52].

Using an acute kidney injury mouse model, Cao et al. [54] concluded that PGI_2_ expression has protective effects against IRI by modulation of the blood flow and control of renin release, resulting in a better restoration of capillary perfusion. In addition, their study described that deletion of PGI_2_ synthesis led to exacerbated IRI and that, in contrast, administration of an exogenous PGI_2_ analog diminished its damage by improving flow control and anti-inflammatory responses.

### 3.2. Vasoconstriction

#### 3.2.1. Thromboxane A_2_ and Cyclooxygenase Enzyme 1

Thromboxane A_2_ (TXA_2_) is also a prostanoid but contradictory to prostacyclin, and it induces vasoconstriction and platelet aggregation. The production of TXA_2_ occurs in a similar pathway to prostacyclin. COX-1 stimulates the isomerization of TXA_2_. In sequence, TXA_2_ binds to receptors in SMCs and platelets, inducing contraction and platelet aggregation, respectively. Vasoconstriction via TXA_2_ can also occur by increasing Ca^2+^ levels in SMCs [48,52]. A balance between PGI_2_/COX-2 and TXA_2_/COX-1 is of utmost importance for maintaining homeostasis. By selectively inhibiting COX-2 in an attempt to reduce inflammation, for example, TXA_2_ will cause vasoconstriction and platelet aggregation without any protection from PGI_2_ activity, therefore accelerating endothelial damage [48,52].

In the clinical setting, renal transplantation has been associated with chronic in vivo platelet activation, thus leading to decreased long-term graft survival. Averna et al. [55] further suggested that this activation is likely a consequence of long-standing endothelial injury that continuously induces platelet activation. In those patients, plasma levels of Willebrand’s factor (vWF) and urinary levels of TXA_2_ metabolites have shown to be an extensively sensitive marker of platelet activation even after several months post-transplant.

#### 3.2.2. Endothelin-1

Glomerular endothelial cells are known to produce endothelin-1 (ET-1), and its receptors are present in both ECs and SMCs. The ET-1 pathway is regulated by the stimulation of inflammatory cells (interleukins and TNF-α) and a reduction in NO and PGI_2_ levels. When SMC ET-1 receptors are activated, there is an influx of Ca^2+^, causing vasoconstriction similar to TXA_2_ activation. In contrast, activation of ET-1 receptors in ECs causes vasodilation by stimulating the release of PGI_2_ and NO. In the event of endothelial dysfunction, EC ET-1 receptors are downregulated, and SMC ET-1 receptors are upregulated, therefore enhancing a vasoconstrictive environment [48,52]. In the transplant setting, clinical studies have reported that vascular ET-1 levels in kidney transplant recipients are markedly increased in renal vessels suffering from transplant vasculopathy and chronic rejection [56].

Additionally, ET-1 goes beyond vasoactive properties. SMC ET-1 receptor binding leads to macrophage activation, neutrophil interaction with the vessel wall, and an increase in free radicals, all of which lead to further endothelial injury. Chronic ET-1 activation can also culminate in SMC proliferation, which increases the thickness of the intima-media in the vessel wall, and it has been associated with the development of atherosclerotic plaques [48,52,57,58]. Several animal transplant and acute ischemic injury studies have shown that administration of (non)selective ET1-antagonists attenuated IRI and organ dysfunction post-transplant, but no studies have implemented such techniques in an ex vivo perfusion model so far [56].

## 4. Vascular (Patho)physiology in the Transplant Setting

Even though transplantation is the best treatment for end-stage renal disease, it is inevitable that the donor graft is exposed to damage—caused by brain death and/or episodes of (warm) ischemia—during the organ retrieval, preservation, and transplant process. This injury can lead to endothelial dysfunction, which in turn can be manifested by reduced vasoactive capacity, activation of hypoxia signaling, inflammation and immune activity, fibrinogenesis, and thrombus formation [33,36]. Subsequently, this acquired injury can lead to complications such as graft primary non-function, shorter graft survival due to acute and/or chronic kidney injury, DGF, and interstitial fibrosis [33]. This section aims to describe and discuss each of these injury processes that take place during acute and chronic transplant-related endothelial dysfunction.

### 4.1. Ischemia-Reperfusion Injury

IRI is a natural and inevitable consequence of transplantation. Disturbances of renal blood flow during arterial clamping, organ procurement—especially in deceased donors—and cold preservation incite multiple episodes of ischemia, leading to tissue hypoxia, ATP depletion, production of reactive oxygen species (ROS), and tissue injury. In addition, while awaiting organ retrieval, a deceased donor is exposed to hormonal changes and resides in an inflammatory and pro-coagulatory state [59]. The last stage of injury occurs during reperfusion, when blood flow and oxygenation are restored, and all the previously inflicted damage is released into the bloodstream. The combination of pre-existing injury within the donor graft, ischemic episodes, and the abrupt re-infliction of blood flow together lead to a subsequent immunological response, possibly leading to a vicious circle of inflammatory signaling and vascular injury [60,61].

IRI has been associated with both tubular and endothelial damage, and the length of cold and warm ischemia time are considered important risk factors. The acute endothelial injury caused by IRI leads to cellular apoptosis and compromises vasoactive capacity. Suboptimal microcirculatory blood flow and cellular injury, in turn, favor activation of coagulation, increased leukocyte adhesion to the vessel walls, and lack of oxygen delivery to the tissue. Irreparable vascular injury culminates in vascular rarefaction, which has been reported to have a tight correlation with renal fibrosis. Significant IRI can manifest as DGF and DGF itself has been associated with decreased long-term graft survival [33].

When looking into direct endothelial-pericyte communication during IRI, a two-pronged effect takes place. First, pericytes are activated, causing them to detach from peritubular capillaries and migrate to the renal interstitium, which further leads to differentiation into myofibroblasts and culminates in fibrosis. Second, this detachment destabilizes endothelial cells, ultimately leading to injury and leakage. This injury contributes to coagulation and inflammation activation, increased oxidative stress, and stimulation of apoptosis [40].

### 4.2. Hypoxia Signaling

The vascular damage caused by the series of ischemic episodes during organ procurement and preservation can contribute to acute kidney injury, which, if unresolved, incites further tissue hypoxia. Hypoxia-related responses are part of vascular homeostasis to avoid injury and supplement repair and regeneration. However, its signaling is not a straightforward process. The heterogeneity extends to inter-individual factors and between different cell types, tissue beds, individual cells within one vascular bed, and even the same cells in different temperature environments [46,62].

One of the most common processes during hypoxia involves the secretion/synthesis of alpha and beta hypoxia-inducible factors (HIFs). In a healthy situation, alpha subunits are degraded with the help of oxygen-dependent prolyl hydroxylases (PHDs) and von Hippel-Lindau protein (VHL). During a hypoxic episode, inhibition of PHD is inhibited, leading to an accumulation of the HIF alpha subunits, which are then free to form a complex with the beta subunit and induces diverse hypoxia responses, such as changes in energy metabolism, tubular epithelial cell repair, cell permeability, and the creation of new vessels [46].

The controlled regulation of blood flow and oxygen delivery between the cortex and the medulla leaves the highly metabolically active cells particularly vulnerable to changes in oxygen supply. The inevitable occurrence of ischemia during transplantation and subsequent IRI has been proven to activate the HIF pathway, which, in turn, protects cells from hypoxic damage by shifting the metabolism from aerobic to anaerobic, therefore facilitating ATP production by using glycolytic activity [63].

HIF-1α stimulation has been suggested to ameliorate vascular and cellular hypoxic injury. However, chronic expression of this alpha subunit can cause hypoxia-induced apoptosis, increased cell permeability, inflammation, and induce predisposition to fibrosis [46,64], meaning that clarifying hypoxic signaling responses in kidneys during preservation, IRI, and machine perfusion is fundamental before designing target therapies to avoid its consequences [46].

### 4.3. Angiogenesis

Another physiological process that is activated after IRI in response to tissue hypoxia and vascular injury is angiogenesis. Kidneys have a very complex vascular structure with many different activities, and the integrity of the vascular bed is important for optimal functioning. When there is damage to the vessels, the organism needs to be able to repair it and restore homeostasis. This process is tightly regulated by a balance between proangiogenic and antiangiogenic factors [65,66,67]. One of the main regulators of endothelial survival, response to hypoxia, migration, division, and sprouting is VEGFs. This cytokine family has different isoforms and expression patterns throughout the kidney: VEGF is present in glomerular and tubular epithelial cells, and VEGF receptors 1 and 2 (VEGFR1/2) are present in peritubular capillaries [65].

During IRI, acute kidney injury affects glomerular and tubular function by inflicting vasoconstriction and inflammation, followed by microcirculation rarefaction and tissue hypoxia, and eventually cell death. Nephron and vascular regeneration are necessary to recover from said damage, but oftentimes, the post-ischemic response is insufficient to regain homeostasis, which contributes to chronic inflammation and interstitial fibrosis [68,69].

Post-ischemic expression of VEGF was investigated in a rat study by Leonard et al. [70], and they showed that suppletion with VEGF-121 immediately after IRI was effective in preserving renal vascular structure compared to delayed treatment and no treatment. However, the exact mechanism of vascular protection was not identified, so it was discussed that perhaps suppletion of VEGF-121 inhibits cell death but has little proliferative effect [68]. An earlier study by Hara et al. [69] further supports this hypothesis by investigating the effects of VEGF inhibition in rats and reporting that blocking VEGF signaling caused increased proteinuria and decreased nephrin.

A porcine transplant study by Shimizu et al. [70] showed that persistent and unresolved inflammation of peritubular capillaries is strongly associated with renal interstitial fibrosis. This finding was later confirmed by the same research group in human kidney graft biopsies, and this inflammatory process was also correlated with post-transplant graft dysfunction and proteinuria [71].

Furthermore, the vasculature is closely related to antibody-mediated rejection. The presence of donor-specific antibodies and human leukocyte antigen (HLA) binding causes endothelial injury that can be measured by elevated tissue protein levels of endothelial transcripts such as von vWF, caveolin-1, platelet/endothelial cell adhesion molecule, and E-selectin. The presence of these markers has been associated with poor long-term graft survival [71]. Persistent inflammation and complement-mediated vascular injury also favor caspase-3 activation, which stimulates the release of extracellular vesicles and culminates in cell death, fibrosis, and vascular rarefaction [33]. Murine and porcine models have shown that inhibiting caspase-3 during renal IRI was associated with better long-term graft survival.

### 4.4. Fibrosis

One of the biggest hurdles in renal transplantation is the shorter long-term survival of deceased donor kidneys in comparison to living donors. Graft failure is most commonly a consequence of chronic allograft nephropathy, which is characterized by a gradual decline in kidney function due to increased interstitial fibrosis and tubular atrophy, meaning that functional units are slowly being replaced by scar tissue. IRI can be a big factor in inciting fibrinogenesis, but relevant amounts of renal fibrosis may also already be present in the donor before transplantation, resulting in less favorable graft quality [20]. Therefore, identifying early-onset fibrosis pre-transplantation and perhaps even inhibiting the chronic activation of its signaling cascade would be of great value.

The key mediator and most studied fibrosis marker is transforming growth factor β (TGFβ). This cytokine, amongst many others, is recruited to the site of injury/inflammation to remove the damaged cells and activate myofibroblasts to induce wound healing. However, when there is chronic inflammation and overstimulation of this cytokine, there is excess deposition of extracellular matrix proteins in the tissue. Over time, this phenomenon affects the function and architecture of the organ [20,72,73].

Studies have been conducted to explore the molecular mechanisms of homeostasis and its impact on endothelial permeability and renal fibrosis, and the sphingolipid signaling pathways have been a topic of interest in recent years. Sphingosine 1-phosphate (S1P) and its receptor S1PR1 have been associated with barrier-protective properties in endothelial cells, and the activation of S1PR1 leads to a redistribution of intercellular junction proteins (VE-cadherin), making them tighter. Using an acute injury mice model with vascular leakage, Akhter et al. [74] demonstrated that, upon endothelial injury, a new population of S1PR1+ cells is generated with high regenerative capacity, therefore restoring vascular integrity [73,74].

### 4.5. Coagulation

In a healthy situation, the endothelium possesses anticoagulant, antithrombic, and fibrinolytic properties. The principal anticoagulant property of the endothelium is via its protein C and protein S receptors, which decrease platelet activation. Platelet aggregation is inhibited by synthetizing prostacyclin and endothelium-derived relaxing factor (EDRF), and endothelial production of heparin-like glycosaminoglycans further inactivates coagulation proteases. Fibrinolysis properties account for fibrin clots by basal synthesis of tissue-type plasminogen activator (t-PA) [36].

Once the endothelium is damaged, a cascade of pro-coagulatory pathways takes place. The exposition of the basal membrane activates vWF and factor XII, which stimulates the extrinsic and intrinsic coagulation pathways, respectively. From then on, glycoprotein receptor IIB IIIA promotes platelet aggregation to other platelets, and platelet degranulation catalyzes the aggregation even further [34,36]. Recent studies have reported that kidney transplantation is associated with an increased risk of thrombotic microangiopathy, which is characterized by endothelial damage and further complement activation. This post-transplant complication affects 0.8–14% of kidney recipients and negatively impacts graft survival [75]. vWF and soluble thrombomodulin levels in renal outflow during reperfusion are potential risk markers for thrombotic complications [76].

## 5. Ex Vivo Perfusion Research in a Transplant Setting

**Figure 3:**
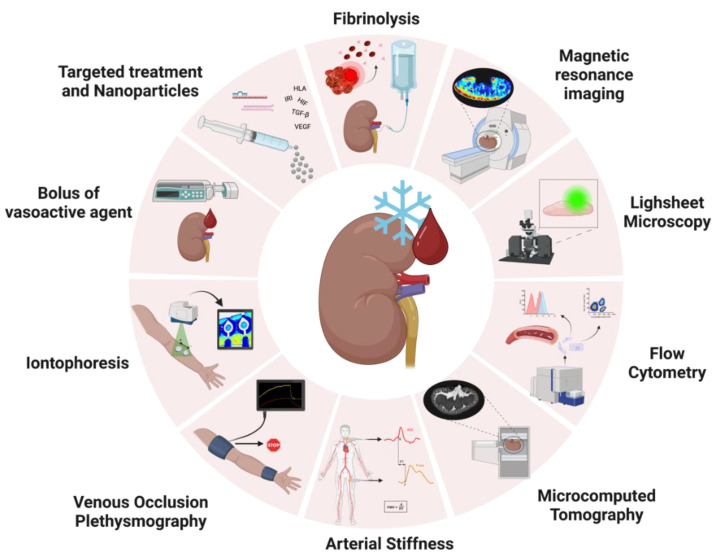
Schematic image of possible applications of vascular viability assessment and targeted treatment from other fields of research in ex vivo perfusion research. Real-time analysis during perfusion could be achieved by administering boluses of vasoactive agents during perfusion, and assessment could be performed by observing flow/vascular resistance variations via ultrasound or laser speckle contrast imaging to observe changes in perfusion profiles; Iontophoresis assesses the nitric oxide availability in the microvasculature by placing two watertight chambers on the skin that conduct an electrical current to transfer positive or negatively charged vasoactive agents through resistance vessels locally. To measure vascular changes, Laser Doppler Flowmetry (LDF) or Laser Doppler Imaging (LDI) can be used; Venous Occlusion Plethysmography entails stopping venous return while there is arterial inflow. This flow occlusion causes the forearm to increase volume over time in proportion to the incoming arterial flow. The increase in forearm volume is measured as the increase in length of the strain-gauge causes a change in electrical resistance; arterial stiffness is measured as branching points in the arteries cause the heartbeat pulsatile pressure waves to be reflected, meaning that the stiffer an artery is, the faster these waves travel back. The most common techniques to assess arterial stiffness are Pulse Wave Analysis (PWA) and Pulse Wave Velocity (PWV); Functional MRI (fMRI) imaging during renal NMP has recently been applied to investigate the impact of relevant events in the transplant process (such as warm/cold ischemia times) on grafts, and it can add an extra set of variables to organ quality prediction models by acquiring information on general vessel architecture and regional blood flow distribution. Sample analysis of vascular viability could be performed by flow cytometry. This technique could be applied to monitor the shedding of endothelial/epithelial cells as a marker of cellular damage; Microcomputed tomography is a relatively new technology that can be applied to image samples inside and out in a non-destructive way and acquire high-resolution images with fast 3D cross-section reconstruction. Voxel sizes can be adjusted to image structures as small as afferent/efferent arterioles; Lightsheet fluorescence microscopy is an ex vivo tissue microscopy technique that enables optical sectioning of the sample while still in a three-dimensional architecture, therefore enabling whole organ imaging. Last but not least, ex vivo perfusion can be used as a platform for targeted treatment, which can be achieved by administering medications (such as drugs, micro RNAs, small interfering RNAs, or even nanoparticles) targeting fibrinolysis inflammation, antibody-mediated rejection (HLA), fibrosis (TGF-β), ischemia-reperfusion injury, hypoxia (HIF), angiogenesis (VEGF). Image created with BioRender.

Given the importance of renal vasculature and the consequences of vascular injury during renal transplantation, it would be extremely helpful if the condition of the renal vasculature could be assessed and potentially improved before transplantation. Ex vivo perfusion, whether it is HMP or NMP, may offer the possibility to assess kidney function prior to transplantation and could provide a treatment platform in a controlled and isolated environment [9,18,20,21,22]. Therefore, investigating and assessing vascular damage during machine perfusion could provide a better understanding of its underlying mechanisms, which could potentially be translated into assessment or treatment techniques. Therefore, we first focus on real-time assessment techniques of the vasculature during ex vivo perfusion. We then highlight several new techniques that could potentially help close the knowledge gap regarding renal vasculature in an ex vivo setting. Last but not least, we explore potential pathways that could be studied and targeted with pharmaceuticals to attenuate vascular damage during machine perfusion. All these techniques and suggestions are represented in Figure 3.

### 5.1. Real-Time Assessment of Vasoactivity during NMP

A healthy endothelium is responsible for modulating the vascular tone according to pressure, flow, and oxygenation conditions. Signals from ECs are received by the SMCs to either relax or contract, therefore creating vasodilation and vasoconstriction. An impaired vasomotor response can be the result of either dysfunction in endothelial signaling to smooth muscle cells or primary dysfunction of SMCs. Therefore, it is important to assess both endothelial-dependent and -independent vasoactive responses [36,48,52]. It would be valuable to explore different techniques known from other fields of research. For example, there are several methods for examining endothelial viability and capillary dysfunction in cardiovascular research [48] that could be implemented in combination with ex vivo perfusion with just some minor adjustments. One of the most common techniques used to assess endothelial-dependent function involves monitoring the vasomotor response to a certain stimulus, while the lack of or diminished response would be an indicator of poor function [48].

#### 5.1.1. Endothelial Vasoactive Response Evaluation

A simple technique that could be implemented during NMP is to administer boluses of vasoactive substances during perfusion and observe flow variations. Those drugs could be directed at a certain vessel layer or vasoactive pathway of interest, and viable vessels should be capable of responding to such stimuli. A more accurate assessment could be achieved by, for example, using ultrasound to measure vessel diameter or laser speckle contrast imaging (LSCI) to observe changes in perfusion profiles [48,77]. In 2018, Bath et al. [19] reported that long warm ischemia times (WIT) directly affect vasodilation capacity after administering vasoactive boluses during NMP, suggesting irreversible endothelial injury, and cold ischemia time (CIT) further diminished that potential. This study indicates that influencing vasoactivity during perfusion could provide a measurement of vascular injury and, therefore, viability.

#### 5.1.2. Iontophoresis

Another technique that could be implemented during NMP is iontophoresis, which assesses the NO availability in the microvasculature [48,78]. While similar to the previously mentioned technique, iontophoresis is a less systemic approach and has not yet been adapted for an ex vivo setting. In the clinic, the principle consists of placing two watertight chambers on the forearm skin that conduct an electrical current to transfer positive or negatively charged vasoactive agents through resistance vessels locally, and the delivery rate depends on the density and strength of the current. Acetylcholine and sodium nitroprusside are the most common agents used in this technique. To measure the vascular changes, laser doppler flowmetry (LDF) or laser doppler imaging (LDI) can be used. LDF and LDI are similar techniques, differing on the size of the scanned area—LDF measures perfusion over a single point, and LDI scans a whole area [48]. With some adaptation and validation, this technique could be implemented during NMP to assess the dynamic vascular response to vasoactive stimuli. In this way, evaluation can be performed in a localized manner without affecting the whole organ, and it would offer real-time results. In addition to doppler techniques, LSCI could be an alternative option for imaging, as it has high special and temporal resolution and has already been validated in perfusion models for microvascular perfusion assessment [77].

#### 5.1.3. Venous Occlusion Plethysmography

Venous occlusion plethysmography could also be applied during NMP with appropriate adaptation/validation. Normally, this technique is used in the forearm, and it entails stopping venous return while there is arterial inflow. This flow occlusion causes the forearm to increase in volume over time in proportion to the incoming arterial flow [48,79]. Two strain gauges are placed high on the forearm and the wrist, one to cause venous occlusion and one to exclude the hand from the measurement. An automated device (plethysmograph) is connected to the forearm cuff and controls the in- and deflation of the gauges respecting the maximum venous filling. The increase in forearm volume is picked up by the machine, as the increase in length of the strain gauge causes a change in electrical resistance, therefore representing an increase in forearm blood flow [48]. In principle, healthy resistance vessels should be able to retain more flow by inducing vasodilation and quickly respond to the strain gauge removal and restore venous return by adjusting vasodilation [79]. This technique could be seen as more aggressive compared to others, but if validated, it could still provide extra real-time information on vascular adaptation capacity during perfusion when testing different protocols or drugs, as it might correlate with other parameters.

#### 5.1.4. Arterial Stiffness

Arterial stiffness is also a principal factor to consider when studying vascular viability [48,80,81]. In vivo, with each heartbeat, pressure waves travel through the vasculature, and compliant arteries dampen these pressure oscillations to deliver blood flow in a smooth manner. At branching points, these pressure waves are reflected, and the stiffer an artery is, the faster these waves travel back. Increased stiffness can be caused by reduced NO production by the endothelial cells, loss of SMC tone, excessive collagen deposition in the vessel wall, and atherosclerosis [48,80]. There are a few techniques available to assess arterial stiffness, the most common being pulse wave analysis (PWA) and pulse wave velocity (PWV), which have been previously associated with coronary microvascular endothelial function [48,82]. Both methods are similar, only differing in the number of measured locations (PWA uses a single point, and PWV uses two points simultaneously). The arterial pressure wave is measured with a transducer by flattening an artery—but not occluding it. Then, the difference between peak readings is calculated as a percentage, and the higher the number, the stiffer the artery. In an NMP setup, a transducer could be easily placed either on the renal artery or on the silicone tubing from the circuit to acquire PWA or PWV data, and arterial stiffness could potentially be measured during perfusion [48]. However, measurement readings would still need to be calibrated and validated for the setup.

### 5.2. Exploring New Techniques to Unravel Molecular Mechanisms of Vascular Injury

Most of the assessment techniques explored above focus on the real-time contractility of the renal vasculature. However, there is still a knowledge gap in general ex vivo physiology, and there are currently no predictive biomarkers for ex vivo renal viability [9]. Since vasculature stability is crucial for maintaining homeostasis and optimal renal function, new techniques should be explored to further understand and investigate its (patho)physiology in an ex vivo manner, as in vivo and ex vivo vascular responses might differ considerably. Although cumbersome, more elaborate and extensive techniques should not be overlooked, as they might be key to unveiling basic ex vivo vascular physiology so that, in the future, they can be simplified and perhaps correlated to more real-time parameters currently in use.

#### 5.2.1. Quantifying Endothelial Cell Shedding with Flow Cytometry

Previous studies have shown that EC damage derived from renal graft injury causes EC shedding, leading to an increase in circulating endothelial cells (cEC) in blood, which correlates with plasma markers such as vWF and E-selectin [83]. During machine perfusion, the presence of cECs could potentially be an indicator of vascular injury. The major advantage of measuring cECs is that their presence is not dependent on EC activation (measured by vWF and E-selectin levels). In that sense, endothelial damage can be differentiated from dysfunction and could therefore aid in graft viability monitoring [83].

Various human and murine studies have used flow cytometry to identify endothelial cells, and a plethora of markers have been studied to identify cells coming from different tissues and vessel sizes [83,84,85,86]. The main antibody that is used to detect cECs in human blood is CD146, but other markers such as CD31, CD144, CD146, CD105, Lectins, VEGF-2, PV-1, vWF, Tie-2/TEK, and HLA-II could be used to phenotype endothelial cells further down to their location within the kidney and vessel size [83,84,85,86]. However, it is important to note that different (donor) kidneys can express these markers in different proportions [85], thus making analysis a complicated process to understand and standardize.

So far, no studies have been performed to investigate the presence of cEC as a vascular viability assessment during machine perfusion. One of the reasons could be that many experimental transplantation studies are performed in pigs due to their similar physiology to humans. Porcine endothelial cells have barely been studied, and the general availability of porcine antibodies is scarce [83]. For flow cytometry analysis, (renal) endothelial-specific pre-labeled primary porcine antibodies are inexistent. Secondly, NMP experiments followed by transplantation are limited due to high costs and little availability of centers for porcine research. As for the clinical situation, quantifying cECs in HMP perfusate is easily applicable as HMP is already standard clinical care. However, since NMP is only barely implemented clinically, flow cytometry during NMP will take some time to initiate. Furthermore, most perfusion studies do not generate post-transplant data as the kidneys are not transplanted, hence correlating cEC numbers with transplant outcomes has not been established.

#### 5.2.2. Lightsheet Fluorescence Microscopy

Lightsheet fluorescence microscopy (LSFM) is an ex vivo tissue microscopy technique, and its imaging principle consists of horizontal fluorescent tissue excitation and vertical scanning [87]. This technique enables optical sectioning of the sample while still in a three-dimensional architecture, therefore enabling whole organ imaging in small animal models. Murine, zebrafish, and organoid models are the most commonly used LSFM during analysis, and their organs of interest vary (hearts, lungs, kidneys, eyes, etc.) [88,89,90,91,92,93,94,95,96]. Several studies have been performed to assess renal vascular viability and architecture using murine models [92,96,97].

During HMP or NMP, the administration of antibodies against endothelial markers—such as CD31, lectins, and glycocalyx components—could potentially help identify the vasculature during LSFM and highlight areas of shedding [85,86,92,96,97]. Additionally, this technique could be used to identify inflammatory activation/infiltration sites along the vessels and overall vascular architecture and even offer insight into capillary rarefaction [97]. As seen in Table 1, the need for tissue fixation limits this technique to retrospective analysis, but it could still be of great help in studying the localized vascular impact of machine perfusion and its potential treatment targets. Since whole porcine and human organs do not fit in the imaging chamber of the microscope, biopsies could be collected and analyzed using LSFM.

#### 5.2.3. Imaging Renal Microvascular Architecture with Microcomputed Tomography

Microcomputed tomography (micro-CT) is a relatively new technology that can be applied to image samples inside and out in a non-destructive way and acquire high-resolution images with fast 3D cross-section reconstruction. This technique has been applied in in vivo studies for the assessment of longitudinal treatment effects [98] and in ex vivo research studies analyzing various organs, such as renal microvasculature architecture imaging and glomerular quantification [96,99]. Voxel sizes can be adjusted to image microvasculature in rodent and porcine models, and structures as small as afferent/efferent arterioles can be visualized. Studies have reported that early structural changes can be detected by micro-CT, and vascular rarefaction, increased microvascular density, and microvascular remodeling have been correlated to early stages of pathological processes such as polycystic kidney disease, hypercholesterolemia, and increased oxidative stress in stenotic kidneys, respectively [96]. In addition, alterations in vascular molecular mechanisms, such as expression of HIF-1α ad VEGF have been associated with changes in vascular structure in polycystic kidneys after micro-CT imaging [96,99].

The advantages of micro-CT, as described in Table 1, are that full geometric structures can be analyzed, glomerular and peritubular capillaries can be distinguished, and the spatial density and tortuosity of vascular beds can be visualized in a relatively fast manner [96,98,99]. However, unlike other imaging modalities, no functional assessments can be performed; this technique requires radiation, and without any modifications to the machine, kidneys cannot be imaged while perfused. Therefore, this technique would be restricted as a post-perfusion analysis in porcine and human discarded kidney models, but observed changes could be correlated to other molecular and/or real-time parameters. In rodent transplantation studies involving ex vivo perfusion as a platform for targeted treatment, micro-CT has the potential to evaluate in vivo vascular response longitudinally for post-transplant monitoring, therefore offering insight into outcomes.

#### 5.2.4. Visualizing Renal Perfusion Profile with Magnetic Resonance Imaging

Magnetic resonance imaging is a technique commonly used in the clinic for non-invasive real-time organ morphological and functional assessment. Several studies published by Schutter et al. [100,101] reveal that functional MRI (fMRI) imaging during renal NMP can be used to unveil renal ex vivo physiology and as a reliable and independent viability assessment tool. The combination of NMP and fMRI could prove of extreme value to investigate the impact of relevant events in the transplant process (warm/cold ischemia times), as well as opening doors to adding an extra set of variables to organ quality prediction models.

During NMP, fMRI acquires information on vascular parameters such as general vessel architecture and regional blood flow. Arterial spin labeling (ASL), for example, is a sequence that tracks inflowing protons while they travel through the kidney, which offers insight into flow distribution [101]. However, as an unexpected result from Schutter’s experiments, ex vivo perfused kidneys showed low cortical flow and high medullary flow for the first two hours, after which it returned to in vivo physiological characteristics. These data highlight once more how different kidneys might behave in vivo and ex vivo. With that in mind, further research into vascular-specific fMRI sequences is needed to investigate and understand renal ex vivo responses, and results should be carefully interpreted.

### 5.3. Treating Vascular Injury in an Ex Vivo Setting

Last but not least, ex vivo machine perfusion can provide a unique opportunity to treat kidney grafts whilst outside of the body. Machine perfusion itself may already offer superior protection against IRI-related vascular damage, but specific drug targeting to repair or prevent additional injury could further improve the preservation of (suboptimal) donor kidneys.

#### 5.3.1. Targeting Hypoxia via the HIF Pathways

An inevitable consequence of ischemia and organ storage is tissue hypoxia, which is most commonly activated via the HIF pathways. With vascular dysfunction and damage, vasoconstriction, inflammation, and thrombosis are phenomena that further lead to lower oxygen delivery to the tissue [46,102]. The HIF signaling cascade is intended to protect the organ from excessive hypoxic damage, but it has been reported that continuous and non-specific activation of HIF pathways can have detrimental consequences. As hypoxia response is very heterogenous even within the same organ—and since the kidney is the most vulnerable to hypoxia damage—it is essential to investigate further the importance of these renal responses in an ex vivo machine perfusion setting.

Several attempts have been made to counteract hypoxia by pre-treating the donor in an experimental setting. Animal studies have reported that pre-treatment of donors by enhancing HIF-1α or inhibiting PHD has a positive effect on post-transplant outcomes, and human observational studies indicated that non-rejected and better-functioning grafts presented with higher HIF-1α levels post-transplant [64,102,103]. However, in a clinical setting, pre-treatment of the donor is very difficult for ethical reasons. Therefore, an ex vivo model could be of significant impact on the ectopic studying and conditioning of kidneys prior to transplantation by directly or indirectly enhancing HIF-1α activity. In 2017, a study by Hollis et al. [64] used renal HMP to deliver PHD inhibitors to investigate whether there was an anaerobic metabolic shift after ischemic injury. Even though a normothermic functional assessment of the kidneys was not performed in this study, the group could show that inhibitor administration during HMP alone could alter the metabolic phenotype of the kidneys. To our knowledge, PHD inhibitors have not yet been tested in a normothermic machine perfusion setup. This would be relevant to examine as PHD inhibitor kinetics could work best under physiological temperatures and, therefore, more protective for the kidney vasculature.

#### 5.3.2. Targeting Angiogenesis via the VEGF Pathway

Another potential vascular treatment pathway is the VEGF pathway. In the face of tissue hypoxia and cellular injury, the vasculature turns to angiogenesis in an attempt to repair its structure and restore homeostasis, and this process is tightly regulated between proangiogenic and antiangiogenic factors [65]. The VEGF pathway has been a focus of various studies in recent years, and enhancement of this pathway in early ischemic processes has shown promise in maintaining vascular viability [68,69]. Once ex vivo angiogenic physiology is unraveled, treatment of kidneys prior to transplantation during perfusion could be an interesting approach to avoid chronic injury. With this setup, similar to HIF pathway interventions, donor treatment is avoided, and the organ would be treated individually without systemic effects. Target agents could be administered momentarily or even associated with microparticles for a longer local effect post-transplant.

#### 5.3.3. Targeting Coagulation with Fibrinolytics

In a healthy situation, the endothelium possesses fibrinolytic, anticoagulant, and antithrombic properties. Damage to ECs negatively impacts their protective effects against inflammation and thrombus formation, making the vascular environment prone to those processes. The exposition of the EC basal membrane activates vWF and factor XII, which stimulates the extrinsic and intrinsic coagulation pathways, respectively. From then on, glycoprotein receptors promote platelet aggregation, and platelet degranulation catalyzes it even further [34,36]. Putting effort into exploring inflammatory and coagulation pathways ex vivo could improve current perfusion protocols to avoid extreme endothelial activation, which can be greatly beneficial for maintaining homeostasis and avoiding further graft damage. As an example, a few previous porcine and human transplant studies have shown that inducing fibrinolysis ex vivo during flush or HMP to actively remove clots from the circulation and prevent further endothelial damage was beneficial for organ functioning, thus improving graft viability post-transplant [104,105].

#### 5.3.4. Using siRNA and miRNA to Attenuate Inflammation

Since endothelial cells are responsible for initiating inflammatory, immunologic, coagulatory, and even graft rejection processes [34,35,36,106], modifying ECs during ex vivo perfusion by reducing or even inhibiting the expression of certain pathways can benefit organ (vascular) viability without further compromising the recipient’s system [107]. This (temporary) cellular reprogramming could possibly be achieved by the administration of small interfering RNAs (siRNA), micro RNAs (miRNA), or even micro/nanoparticles loaded with certain therapeutic agents [108,109].

A study by Cui et al. [108], for example, pre-treated human epicardial coronary arteries with siRNA-loaded biodegradable poly(amine-co-ester) (PACE) nanoparticle against class II transactivator (CIITA) via NMP. CIITA is a positive regulator for the transcription of class II major histocompatibility complex (MHC-II) molecules, and it induces temporary unresponsiveness to interferon (IFN)-γ-mediated induction of MHC-II molecules. Isolated arteries were perfused for 6 hours at 37 °C with PACE nanoparticles resuspended in M199 medium. Afterward, these vessels were transplanted into the infra-renal aorta of immunodeficient SCID/beige mice and monitored for 6 weeks. After 1 week, nanoparticles were not detected in other mouse organs, and after 6 weeks of transplant, pre-treated arteries still showed some level of MHC-II expression inhibition. Moreover, treated arteries retained a more favorable endothelial cell coverage, smooth muscle cell function, and reduced T-cell infiltration. This study also reported that the flow rate during perfusion is an important factor for optimal distribution and kinetics of the nanoparticles, as their association decreased in the face of increased shear stress.

Micro RNAs could be used as a therapeutic target by administering miRNAs systemically or locally to either inhibit or enhance certain transcript functions. Their efficacy has been reported in studies targeting cancer, hepatitis C, heart abnormalities, kidney disease, pathologic fibrosis, etc. [109]. Administration of certain miRNAs during ex vivo perfusion could be beneficial in more than one way. Firstly, ex vivo perfusion would serve as a pre-transplant treatment platform to prevent excessive damage or enhance repair. Secondly, it would be a platform to deliver such treatments in an isolated manner, therefore avoiding any systemic responses post-transplant.

#### 5.3.5. Targeting Fibrosis via the TGF-β Pathway

The last step of renal injury is fibrosis. This process is the result of irreparable damage to the vasculature and renal tissue, in which TGF-β plays a key role. Attenuating fibrosis during renal ex vivo perfusion could be an interesting approach to prevent its onset due to persistent inflammation after IRI and to allow the vasculature to repair and re-vascularize prior to the no-return point. Avoiding vascular injury and rarefaction is crucial to avoiding further fibrinogenesis.

Drugs to target such pathways could be easily administered in an HMP or NMP perfusate [20]. However, fibrosis is a slow process that cannot be observed within only a few hours, so other techniques need to be explored in addition to machine perfusion alone. Recent studies by van Furth et al. [110] and van Leeuwen et al. [20] have proposed the use of precision-cut kidney slices as a model for the long-term evaluation of drug effect and IRI molecular mechanisms. This model has the benefit of being able to use a single organ to test various drugs simultaneously, and it is also an upgrade from cell culture as it maintains part of the tissue’s architecture, therefore allowing evaluation of cell-cell and cell-matrix interactions. Using this technique after administering drugs during ex vivo perfusion could open doors to investigating long-term effects (24–48 h) of fibrosis in renal tissue. Ultimately, they have shown that fibrosis can be attenuated by targeting the TGF-β pathway.

## 6. Conclusions

Vascular complications play a prominent role in transplant-related short- and long-term function. Better understanding, assessing, and perhaps even treating the renal vasculature pre-transplant would offer great potential for improving transplant outcomes. Maintaining vascular homeostasis is crucial for renal cellular survival and organ function, and dysregulation of this process presents interconnected short- and long-term impacts on viability. Organ preservation research has improved and grown exponentially in recent years, and ex vivo perfusion could act as an intermediate step between donor organ procurement and recipient surgery to assess viability and improve graft quality. Reliable, independent biomarkers or assessment techniques for renal viability are still to be unveiled, and although the vasculature is the starting point for multiple dysfunction processes and graft rejection, it is still the least researched renal compartment in the spectrum of machine perfusion.

Current vasculature-related assessment parameters such as vascular resistance, flow, and urine production lack significance in predicting optimal ex vivo renal function. Therefore, taking a step back into basic physiology research and exploring new treatments and technologies—perhaps applied in different fields of research—could help fill that gap. Even if restricted to an experimental setting, more elaborate and extensive techniques should be explored, as they might offer insights into basic ex vivo vascular physiology so that in the future, they might be simplified and perhaps correlated to more real-time parameters currently in use. Despite the lack of specific literature, the techniques and treatments discussed in this review could potentially enhance post-transplant outcomes and graft survival and ultimately improve the transplant recipients’ quality of life.

## Figures and Tables

**Table 1:** Summarized view of previous applications and (dis)advantages of suggested techniques.

Technique	Previous Application	Advantages	Disadvantages
Response to vasoactive stimuli	Cardiovascular research and ex vivo perfusion	Real-time evaluation	Affects the whole organ
Iontophoresis	Clinically used	Real-time evaluation; local effect	Requires validation and placement of chambers on the kidney surface
Ultrasound/Laser doppler	Clinically used	Already clinically used; Real-time evaluation;	No generalized assessment possible; Microvasculature not visible; Requires placement of probe on kidney surface;
Laser speckle imaging	Ex vivo perfusion	Real-time evaluation; Already validated during ex vivo perfusion; microvasculature more visible	Needs a specific dark chamber for scanning; Only superficial visualization is possible
Venous occlusion plethysmography	Clinically used	Real-time evaluation; No need to administer drugs	More aggressive; Affects the whole organ by occlusion of outflow
Arterial stiffness	Clinically used	Real-time evaluation; No need to administer drugs; Could possibly be measured in the tubing	Requires validation and possible placement of probes on the kidney surface
Flow cytometry	Human, murine studies	Specific analysis/labeling of desired cells/proteins	Not a real-time assessment; Antibody availability could be difficult;
Lightsheet microscopy	Murine, zebrafish, organoid studies, etc.	Three-dimensional analysis of sample; Specific analysis/labeling of desired cells/proteins	Not a real-time assessment; Not possible to do whole organ analysis in large animal/human models; Sample needs to be fixed and cleared; Lengthy
Microcomputed tomography	Murine in vivo and ex vivo	Three-dimensional analysis of sample; Possible to visualize small structures; Not lengthy; In vivo scanning in small animal models	Not a real-time assessment; Not possible to perform functional analysis
Magnetic resonance imaging	Clinically used and ex vivo perfusion	Possible to perform anatomical and functional analysis; Already applied to ex vivo perfusion; Real-time assessment	Still under study for interpretation during ex vivo perfusion; Logistically cumbersome
Targeted treatment and nanoparticles	Human, porcine, and murine studies, ex vivo perfusion	Treatment is delivered to the isolated organ; Nanoparticles allow long-term drug delivery	No long-term assessment is possible during ex vivo perfusion; Drug dosage needs validation and tested for toxicity
siRNA and/or miRNA treatment	Cardiovascular research, murine and human studies, and ex vivo perfusion	Treatment is delivered to the isolated organ; Treatment can have an effect even after transplantation	No long-term assessment possible during ex vivo perfusion; Drug dosage needs validation and tested for toxicity
Precision-cut slices	Murine, porcine, and human studies, ex vivo perfusion studies	Long-term assessment possible; Multiple drugs can be tested in a single organ	Full organ functionality not possible; Prone to infections
Fibrinolysis	Human and porcine transplant studies, ex vivo perfusion	Treatment is delivered to the isolated organ; Avoids further graft damage	Post-transplant consequences need more extensive study

## Data Availability

Not applicable.
